# Supplementary material for: Evaluating PI-RADS lesions and clinically significant prostate cancer in Black and Asian men: a PREVENT randomized clinical trial secondary analysis
Source: Prostate Cancer Prostatic Dis. 2025 Nov 26;29(2):435–9. doi: 10.1038/s41391-025-01057-5 (PMC13190335; doi:10.1038/s41391-025-01057-5)

Supplementary material

Supplementary Methods 1: Original PREVENT Trial Protocol
Supplementary Table 1: Collaborators by Institution
Supplementary Table 2: Investigator Biopsy Experience and Biopsy Technique
Supplementary Table 3: Details on Infection Adverse Events
Supplementary Table 4: Details on Non-Infection Adverse Events
Supplementary Table 5: Details on Protocol Violations
Supplementary Table 6: Accruals by type of consent
Supplementary Figure 1: Original PREVENT Trial CONSORT Diagram

Supplementary Methods 1. Original PREVENT Trial Protocol

The trial protocol was approved by the institutional review board at study sites and Biomedical Research Alliance of New York 18-02-365. The trial biostatistician (M.A.) analyzed the data. No commercial entity was involved in the trial. The trial was funded by the National Cancer Institute, which was not involved in protocol development, data analysis or interpretation, or manuscript preparation. Deviations were monitored by the New York Presbyterian Weill Cornell Medicine (NYP-WCM) Data Safety Monitoring Committee.

We assumed one infection (0.4%) in the transperineal arm and a 5% infection risk in the transrectal arm. We estimated that randomization with 236 patients per arm would provide 80% power to reject the null hypothesis of no difference in infections, given a two-sided α of 0.05. The sample size was increased to 530 to allow for 10% withdrawal and loss to follow-up.
From March 2021 through May 2023, patients were recruited at ten centers and were eligible for enrollment if they had not undergone prior prostate biopsy, had an elevated PSA level and/or abnormal digital rectal examination, and had suspicious prostate magnetic resonance imaging (MRI) characteristics (Prostate Imaging Reporting and Data System, version 2.1 scores 3–5). A small number of participants had biopsy without MRI (claustrophobia and metal prosthesis) and were included as our primary outcome was infection. The exclusion criteria included acute prostatitis in the last 6 mo or any current bacterial infection requiring antibiotic treatment. There have been no significant methodological (eligibility or outcomes) changes following trial commencement.

We used two-stage informed consent, although in some sites performing predominantly transperineal biopsy, traditional one-stage consent was used. Research coordinators implemented the assignment sequence, which used randomly permuted blocks of unequal size stratified by the urologist and PSA (<4, 4–9.9, and ≥10 ng/ml), through central password-protected, web-based randomization, ensuring that allocation could not be predicted before or modified after randomized. Allocation was not blinded to researchers or participants.
A rectal culture to screen for fluoroquinolone-resistant organisms was obtained prior to transrectal biopsy, and targeted antibiotic prophylaxis was administered. In the absence of resistance, fluoroquinolone prophylaxis was administered; an alternative based on culture sensitivities was used for participants allergic to fluoroquinolones. In the 15% of participants with rectal culture–fluoroquinolone resistance, an alternative antibiotic was given based on sensitivities. No antibiotic prophylaxis was given for transperineal biopsy. Investigators followed the trial protocol–specified biopsy procedural approach and used the transperineal local anesthetic approached described by Kubo et al. A maximum of 20 ml of 1% lidocaine was used for both approaches, with 12 systematic biopsy cores and three targeted cores per MRI region of interest.

Immediately after biopsy, participants completed a numerical rating scale (0–10) assessment of pain, discomfort, and anxiety, with higher scores indicating greater symptom intensity.
In addition, we ascertained outcomes of interest through 7-d, because all infections, hospitalizations, and 99.9% postbiopsy adverse events requiring intervention (Common Terminology Criteria for Adverse Events v.5.0 of grade ≥2) occur within this time frame. Adverse events were followed until resolution. Infectious complications included uncomplicated urinary tract infection (UTI), complicated UTI, and urosepsis.

The secondary outcomes included bleeding requiring intervention and urinary retention captured through a combination of a prospective medical review and the 7-d survey. The 7-d survey comprised the general symptom items from the validated biopsy TRUS-BxQ instrument. Items queried for fevers, chills, UTIs, acute urinary retention, new medications, additional post biopsy healthcare encounters, and persistent pain and discomfort. If participants reported additional encounters, they were contacted to rule out adverse events treated at an outside facility. Healthcare providers were also queried regarding adverse events when reviewing biopsy pathology. We also compared the detection of clinically significant (grade group 2–5) and low-grade (grade group 1) cancer.

The primary analyses followed the intent-to-treat principles. We also performed planned per-protocol analyses by type of biopsy received after excluding 26 participants with protocol deviations. Multivariable logistic regression with site as a covariate was used to analyze the association between the randomization arm and infection, urinary retention, bleeding requiring intervention, and detection of clinically significant and insignificant cancer. Adjusted absolute risk differences were calculated using regression least-squares adjusted mean difference. However, due to the small number of significant adverse events, we were unable to adjust for site, and instead used Fisher's exact test and estimated the difference in proportions along with the Newcombe hybrid score 95% confidence intervals (CIs).

We tested for a difference in biopsy pain, discomfort, and anxiety at biopsy, and pain and discomfort were compared from the 7-d survey. Categorical responses were analyzed as described above, and continuous responses were analyzed using the analysis of covariance with randomization strata as covariates. All analyses were conducted using R version 4.2.2 with the tidyverse (v1.3.2), gtsummary (v1.6.3), and emmeans (v1.8.4.1) packages.

Supplementary Table 1. Collaborators by Institution.

Institution	Consent approach	Authorship/Acknowledgments
Weill Cornell Medicine, New York City, NY	Two-stage	Jim Hu, Chunmei McKernan, Sheri Cheng, Bouchra Benghomari, Siwen Xie, Thomas Flynn, Miko Yu
Northwestern Medicine, Chicago, IL	Two-stage	Edward Schaeffer, Ashley Ross, Shilajit Kundu, Hiten Patel, Anthony Schaeffer, Teresa Zembower, Margarita Mana-ay, Nikki Hubbard
Johns Hopkins Medicine, Baltimore, MD	One-stage	Mohammad Allaf, Andrew Cohen, Christian Pavlovich, Misop Han, Michael Rezaee, Claire de la Calle, Kyle Spradling, Ahmed Ghazi, Nirmish Singla, Eric Katz, Rana Harb, Tina Wlajnitz, Johnathan Shepard, Julia Faranetta
Memorial Sloan Kettering, New York City, NY	Two-stage	Behfar Ehdaie, Mireya Arango
University of Connecticut Health, Farmington, CT	One-stage	Benjamin T. Ristau, Melanie Klinck, Maria Clemencia Ortiz, Jillian McNamara, Katarzyna Nastri, Quratulain Ali, Vincent Rella, Madison Lowe, Juliette Dudek
University of Michigan	Two-stage	Jeffrey Montgomery, Sharada Lanka
MedStar Health at Georgetown University School of Medicine, Washington D.C.	One-stage	Keith Kowalczyk, Diana Shmul
University Health Hospital at Case Western Reserve University School of Medicine, Cleveland, OH	Two-stage	Jonathan Shoag, Lydia Beard, Adam Calaway

Commented [VA1]: Need to add NYP queens and brooklyn

Supplementary Table 2. Investigator self-reported biopsy experience by approach and trial biopsy volume.

Investigator	Transrectal biopsy			Transperineal biopsy			Total Trial Volume
	Approach and Platform	Prior Experience	Trial Volume	Approach and Platform	Prior Experience	Trial Volume	
Jim Hu	Artemis software fusion	1500	112	PrecisionPoint fusion*	200	124	236
Edward Schaeffer	UroNav software fusion	2000	67	PrecisionPoint fusion	150	71	138
Mohammad Allaf	UroNav software fusion	1200	16	UroNav software fusion	500	13	29
Behfar Ehdaie	Koelis software fusion	2000	18	Koelis software fusion	500	7	25
Andrew Cohen	Cognitive fusion	300	1	PrecisionPoint fusion	75	24	25
Benjamin Ristau	Cognitive fusion	180	12	Cognitive fusion	120	9	21
David Green	Cognitive fusion	400	8	PrecisionPoint fusion	100	9	17
JHM investigators†	Artemis software fusion	unknown	6	PrecisionPoint fusion	unknown	5	11
Michael Rezaee	UroNav software fusion	300	8	UroNav software fusion	40	3	11
Misop Han	UroNav software fusion	3000	9	UroNav software fusion	50	2	11
Christian Pavolvich	UroNav software fusion	2000	5	UroNav software fusion	600	3	8
John Graham	Navigo software fusion	200	4	PrecisionPoint fusion	20	3	7
Jeffrey Montgomery	UroNav software fusion	800	5	UroNav software fusion	80	1	6
Ashley Ross	UroNav software fusion	1000	2	PrecisionPoint fusion	25	3	5
Keith Kowalczyk	Cognitive fusion	500	3	PrecisionPoint fusion	200	2	5
Shilajit Kundu	UroNav software fusion	300	1	PrecisionPoint fusion	100	3	4
Patel Hiten	UroNav software fusion	60	2	UroNav software fusion	10	1	3

Gerald Wang	Artemis software fusion	600	0	PrecisionPoint fusion	100	2	2
Jonathan Shoag	UroNav software fusion	100	0	UroNav software fusion	200	1	1
Ahmed Ghazi	UroNav software fusion	500	0	UroNav software fusion	150	1	1
Nirmish Singla	UroNav software fusion	150	1	PrecisionPoint fusion	8	0	1

UroNav Phillips; Andover, MA; Artemis Eigen; Grass Valley, CA; Koelis; Princeton, NJ; PrecisionPoint Perineologic; Cumberland, MD; Navigo UC-Care; Yokne'am Ilit, Israel

*PrecisionPoint fusion denotes cognitive fusion.
† Johns Hopkins Medicine investigators: Claire de la Calle, Kyle Spradling and Eric Katz left the institution.

Supplementary Table 3. Details of Infectious Complications.

Subject	Rectal culture Findings	Prophylaxis	Presentation	Laboratory Results
1	Sensitive to fluoroquinolone	ciprofloxacin	Fever to 102<br>Hospital admission through ER	Urine culture demonstrated $>10^5$ E. Coli sensitive ciprofloxicin<br>Blood culture negative
2	Sensitive to fluoroquinolone	ciprofloxacin	Fever to 102 seen by PCP	Urine culture negative<br>WBC 15.8,<br>Urine dip WBC 2+
3	Resistant to Fluoroquinolone, sensitive to cefpodoxime	cefpodoxime	Fever to 103<br>Treated with IV hydration and antibiotics in ER	Urine culture demonstrated $>10^5$ E. Coli, sensitive to fluoroquinolone
4*	Resistant to fluoroquinolone	trimethoprim-sulfamethoxazole<br>ceftriaxone	Fever to 104<br>Hospital admission through ER	Urine culture 10000-49000 CFU resistant to ciprofloxacin and ceftriaxone<br>Blood culture E Coli resistant to ciprofloxacin and ceftriaxone

*Protocol violation because rectal swab culture did not yield sensitivity and resistance patterns.
PCP= primary care physician
CFU= colony forming units

Trial Definitions of Infectious Complications

Uncomplicated UTI:
1. Symptoms of dysuria, urgency, frequency, or hematuria
2. Pyuria and/or bacteriuria
3. No fever

Complicated UTI:
1. Symptoms of fever, flank pain, nausea/vomiting
2. Pyuria and/or bacteriuria

Sepsis:
1. Meets criteria for sepsis, severe sepsis, or septic shock
2. Evidence of urinary pathogen growth in urine or blood cultures

Supplementary Table 4. Non-Infectious biopsy Adverse Events.

Bloody diarrhea over 2 days	Participant seen in the emergency room six days after transrectal prostate biopsy and CT scan revealed extravasation in the rectal region. Colonoscopy demonstrated a small l clot over the recent biopsy site, and a clip was placed. No transfusion was needed, and he was discharged after a two-day hospitalization for a CTCAE grade 3 adverse event.	Hematocrit 35.1
Urinary retention	Foley catheters were placed in the 3 transrectal and 1 transperineal biopsy patients, who could not urinate within four days of prostate biopsy. All passed voiding trials after 7-days for these CTCAE grade 2 adverse events.	NA

Supplementary Table 5. Protocol violation details by subject and site.

Site	Randomization	Biopsy Approach	Protocol Violation
NYP WCM	Transperineal	Transrectal	Received sulfamethoxazole-trimethoprim and ceftriaxone
NYP WCM	Transrectal	Transrectal	Received sulfamethoxazole-trimethoprim and ceftriaxone
NYP WCM	Transperineal	Transperineal	Received levofloxacin
NYP WCM	Transrectal	Transrectal	Received ceftriaxone; no resistance on rectal swab
NYP WCM	Transrectal	Transrectal	Received sulfamethoxazole-trimethoprim for no resistance on rectal swab
NYP WCM	Transperineal	Transperineal	Received levofloxacin
NYP WCM	Transperineal	Transperineal	Received levofloxacin
NYP WCM	Transperineal	Transperineal	Received levofloxacin and sulfamethoxazole-trimethoprim
NYP WCM	Transperineal	Transperineal	Received levofloxacin
NYP WCM	Transrectal	Transrectal	Received levofloxacin and gentamicin; no rectal swab results
NYP WCM	Transrectal	Transrectal	Received sulfamethoxazole-trimethoprim; no resistance on swab
NYP WCM	Transrectal	Transrectal	Received ceftriaxone; no resistance on rectal swab
NYP WCM	Transrectal	Transrectal	Received ceftriaxone; no resistance on rectal swab
NYP WCM	Transrectal	Transrectal	Received gentamicin and levofloxacin for negative swab
NYP WCM	Transrectal	Transrectal	Received ceftriaxone; no resistance on rectal swab
NYP WCM	Transrectal	Transperineal	Received levofloxacin and gentamicin; no rectal swab
Johns Hopkins	Transperineal	Transrectal	Received levofloxacin
Johns Hopkins	Transperineal	Transrectal	Received gentamicin
Johns Hopkins	Transrectal	Transperineal	Received minimal sedation
Johns Hopkins	Transperineal	Transperineal	History of prior negative biopsy
Johns Hopkins	Transperineal	Transperineal	Received ceftriaxone
NYP Brooklyn	Transrectal	Transrectal	Received ceftriaxone; no rectal swab
NYP Brooklyn	Transrectal	Transrectal	Received ceftriaxone; no resistance on rectal swab
NYP Brooklyn	Transrectal	Transrectal	Received gentamicin; no resistance on rectal swab
NYP Brooklyn	Transrectal	Transrectal	Received ceftriaxone; no resistance on rectal swab
NYP Brooklyn	Transrectal	Transrectal	Received ceftriaxone; no resistance on rectal swab

Supplementary Table 6. Accrual by type of consent.

**Type of consent**	**Number of accruing urologists**	**Total accrual**	**Accrual per urologist**
Two-stage	12	473	39.4
One-stage	9	94	10.4

Supplementary Figure 1. CONSORT diagram showing the flow of participants through various stages of a randomized trial. MRI = magnetic resonance imaging.

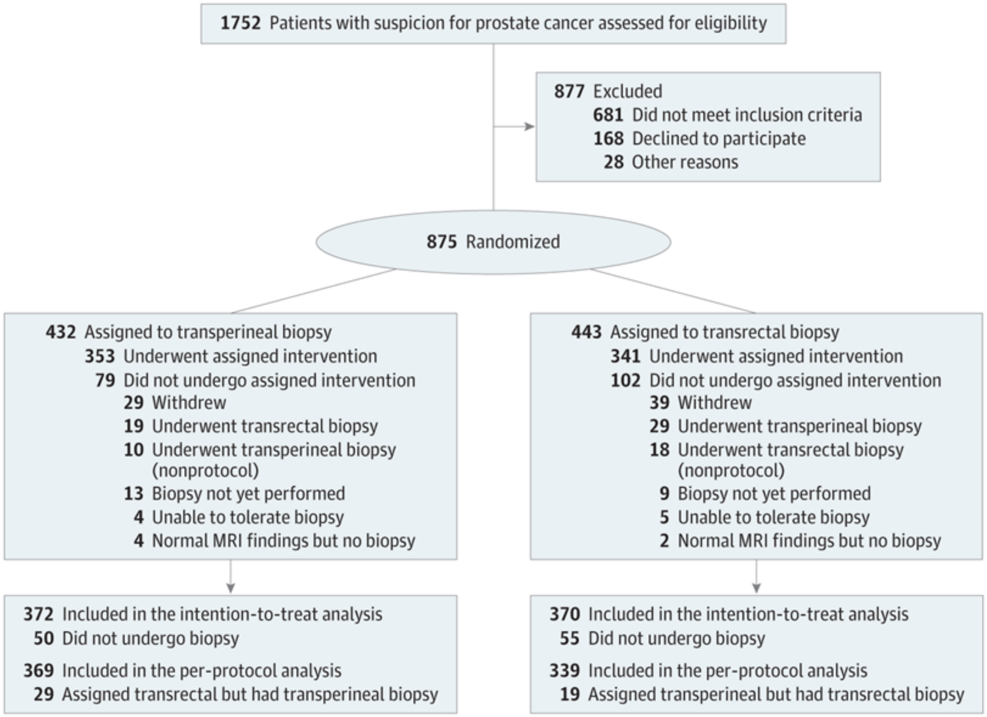

Supplement: Supplementary file 1 — Supplementary Material [file 41391_2025_1057_MOESM1_ESM.pdf]
